# Predicting the demand of physician workforce: an international model based on "crowd behaviors"

**DOI:** 10.1186/1472-6963-12-79

**Published:** 2012-03-26

**Authors:** Tsuen-Chiuan Tsai, Misha Eliasziw, Der-Fang Chen

**Affiliations:** 1 Department of Pediatrics, E-Da Hospital, No.1, Yida Road, Jiaosu Village, Yanchao District, Kaohsiung City 82445, Taiwan, Republic of China; 2 Department of Chinese Medicine, I-Shou University College of Medicine, No.1, Yida Road, Jiaosu Village, Yanchao District, Kaohsiung City 82445, Taiwan, Republic of China; 3 Departments of Community Health Sciences, Clinical Neurosciences, Oncology, University of Calgary, 2500 University Dr. NW, Calgary, AB T2N 1 N4, Canada; 4 Department of Surgery, Cathay General Hospital, No.280, Sec. 4, Ren'ai Rd., Da'an Dist, Taipei City, Taiwan, Republic of China; 5 Fu-Jen Catholic University College of Medicine, No.280, Sec. 4, Ren'ai Rd., Da'an Dist, Taipei City, Taiwan, Republic of China

**Keywords:** Physician manpower, Medical education, Healthcare quality, Physician demand, Prediction model

## Abstract

**Background:**

Appropriateness of physician workforce greatly influences the quality of healthcare. When facing the crisis of physician shortages, the correction of manpower always takes an extended time period, and both the public and health personnel suffer. To calculate an appropriate number of Physician Density (PD) for a specific country, this study was designed to create a PD prediction model, based on health-related data from many countries.

**Methods:**

Twelve factors that could possibly impact physicians' demand were chosen, and data of these factors from 130 countries (by reviewing 195) were extracted. Multiple stepwise-linear regression was used to derive the PD prediction model, and a split-sample cross-validation procedure was performed to evaluate the generalizability of the results.

**Results:**

Using data from 130 countries, with the consideration of the correlation between variables, and preventing multi-collinearity, seven out of the 12 predictor variables were selected for entry into the stepwise regression procedure. The final model was: PD = (5.014 - 0.128 × proportion under age 15 years + 0.034 × life expectancy)^2^, with R^2 ^of 80.4%. Using the prediction equation, 70 countries had PDs with "negative discrepancy", while 58 had PDs with "positive discrepancy".

**Conclusion:**

This study provided a regression-based PD model to calculate a "norm" number of PD for a specific country. A large PD discrepancy in a country indicates the needs to examine physician's workloads and their well-being, the effectiveness/efficiency of medical care, the promotion of population health and the team resource management.

## Background

Physicians are the key personnel who make medical decisions and deliver medical treatments to patients. The adequacy of a country's physician workforce greatly influences the quality of healthcare. The literature indicated growth in health worker density significantly reduced the burden of disease, especially the burden associated with communicable diseases [1]. On the contrary, physician shortages translated into inadequate care [2,3] and greater costs for the treatment of disease [4,5]. However, physician size has been reported not always positively related with healthcare quality. Physicians may induce demands, and physician surpluses may drive unnecessary utilization of healthcare [6]. As the rapid progression of globalization, physician migration across country borders has become more intense than ever [7]. During the past years, there have been 384 citations with the tag of "physician supply and/or demand" [8]. Appropriately matching physician supply and demand is now a critical worldwide concern.

Estimating the number of physicians a country requires is a complex task given the many contributing factors that have impacts on physicians' productivity, people's expectation of healthcare quality and the utilization of healthcare resources [9,10]. These factors are theoretically divided into four domains: population, physician, healthcare system, and economics. Within the population domain, one needs to consider the age, birth rate, death rate, infant mortality, life expectancy, population growth rate, incidence and prevalence of diseases, health demands by age, literacy, and health expectations. Within the physician domain, it is necessary to take into account the practicing physician's age (as a measure of the number of physicians retiring), gender, specialty and subspecialty, number of work hours per week, and clinical competence. Within the healthcare system domain, there is a need to consider healthcare accessibility, number of hospital beds, availability of resources, structure of payment, availability of support personnel (i.e., nurses, midwives, and technicians) and overall treatment capacities. Final considerations need to be on the economics of a country, often expressed as GDP (gross domestic product), GNP (gross national product), GNPPC (GNP per person), or PPP (Purchasing Power Parity). The above factors may be correlated with the size of a country's physician workforce, but not necessarily have a causal relationship. As population size is known to be an essential factor that determines physician demand, physician density (PD, defined as the number of practicing physicians per 10,000 population) is often used to estimate physician needs.

Upon reviewing the literature, many factors have been reported to have significant relationships with physician density. Using a regression analysis of the World Bank data on 250 countries, physician density was reported to be influenced by GDP, female literacy, the percentage of female population aged over 60 years, and female life expectancy [10]. Similarly, a regression analysis for both LMICs (Low and Low-Middle Income Countries) and MHICs(middle and high income countries) showed that physician density was significantly associated with several health indicators of infant mortality rate, under 5 (year of age) mortality rate, maternal mortality rate, and life expectancy [1]. The number of hospital beds (per 1,000 inhabitants) had a positive impact on the growth rate of specialists [6], and the disability-adjusted life years (DALYs) had a significantly inverse relationship with the density of health workers [11]. As the above factors were observed to interact with one another, and with other hidden factors (e.g., economic factors underlying the child population factor), added by the difficulty in measurement (e.g., physician competency), the prediction of manpower for future needs became very difficult, especially in a rapidly changing health care environment. Currently, there has been no tool or formula that can accurately predict the optimal PD for a given country.

To predict reality, "the Wisdom of Crowd theory", suggested to aggregate information in "groups" rather than in "individual" [12]. In the book, entitled "*The Wisdom of Crowds"*, Surowiecki argued that aggregating information in groups results in predicting reality better than by a single members of the groups. The opening anecdote in the book described "Francis Galton's surprise that the crowd at a country fair accurately guessed the weight of an ox; when the individual guesses were averaged; the average was closer to the true butchered weight of the ox than the estimates of individual crowd members."

With the rapid growth of information technology, abundant data on health indices and population demography has accumulated across country boundaries. Using aggregated international data of "crowd behaviors", the aim of the present study was to develop a PD prediction model for evaluating the needs of physician manpower that closely reflects the reality. When calculating the discrepancy between the observed and the predicted PD, the model may be used to screen the appropriateness of physician manpower in a nation, and provide warnings to prevent from the damage to healthcare in its early stage.

## Methods

Twelve variables that were readily accessible on the world wide web, and having a possible impact on physicians' demands, were chosen. The variables consisted of health indicators, population demography, health care system, and socioeconomic status. The indicators were under age 5 year mortality rate, adult mortality rate, life expectancy, fertility rate, literacy, population density, proportion under age 15 years, proportion over age 60 years, gross domestic product, gross national income, purchasing power parities, and expenditure on health.

Data on PD and 12 variables were extracted from the World Health Organization (WHO), United Nations (UN), Organization for Economic Cooperation (OECD), and World Bank (WB) data banks for the years of 2004, 2005, and 2006. The study received ethical approval from IRB (Institutional Review Board) in Wanfang Hospital Taipei Medical University, with the reference number of 97044.

### Statistical analyses

A six-step procedure was used to derive the international prediction model for PD, which consisted of: 1) reducing data by eliminating highly correlated variables, 2) selecting countries with complete data in the analysis, 3) dividing the countries randomly into two halves, 4) generating a prediction equation from the first half of the countries and using it to predict the observed PD for countries in the second half, 5) subsequent to the split-sample validation in step 4, the countries were combined into one dataset to derive an overall international PD model, and 6) the model was then used to predict PD and to calculate country-specific PD discrepancies.

Multiple stepwise-linear regression was used to derive the model that best predicted PD. A p-value of 0.15 was used for both the variable entry and variable removal criteria [13]. The sample squared multiple correlation (R^2^) was used to quantify the strength of the relationship in terms of the percentage of data variation explained by the regression model. Adequacy of model assumptions were assessed by a normal probability plot and by a plot of standardized residuals versus predicted values.

For the purpose of analyses, the mean PD across the 3 years (2004, 2005, and 2006) was calculated to be the outcome variable as the Pearson correlation (r) among the years was high (r > 0.97). Similarly, country-specific predictors were calculated as the mean of sex-specific and year-specific data because the correlations were also high (r > 0.96). Literacy was excluded from all the analyses because information was available only on half of the countries. Among the 11 remaining predictors, several were observed to be highly correlated with each other: under age 5 years mortality rate, adult mortality rate, and life expectancy (r > 0.93), proportion under age 15 years and fertility rate (r > 0.94), gross domestic product and gross national income (r > 0.94). As a result, the following 4 predictors were excluded from all analyses to prevent multi-collinearity: under age 5 years mortality rate, adult mortality rate, fertility rate, and gross national income. The remaining 7 predictor variables were considered for entry into the stepwise regression procedure: population density, proportion under age 15 years, proportion over age 60 years, life expectancy, gross domestic product, expenditure on health, and purchasing power parities.

A split-sample cross-validation was performed to assess the generalizability of the results [14]. The process consisted of splitting the original sample into a training set and validation set using random sampling. A regression equation was derived in the training set and the R^2 ^between the observed and predicted response values was calculated. The regression coefficients from the training set were then used to calculate predicted values in the validation set. The cross-validation coefficient (R^2^*) between these predicted values and observed values in the validation set was calculated. The shrinkage coefficient was calculated as the difference between R^2 ^and R^2^*of the training and validation sets. The smaller the shrinkage coefficient, the more confidence one can have in the generalizability of the results. Shrinkage coefficient values less than 5% indicate a generalizable model [14]. Given a satisfactory shrinkage coefficient, the data were combined from both sets and a final regression equation was derived based upon the entire sample. The final model was then applied to all the countries to calculate country-specific PD discrepancies (predicted PD minus observed PD) and the predicted number of required physicians using 2009 country populations.

The countries were then stratified by area and economic status. Analysis of covariance (ANCOVA), adjusting for observed PD, was used to examine whether the country-specific PD discrepancies differed by continent, membership in the OECD (Organization for Economic Cooperation and Development), and by economic status (low income, middle income, high income). Least squares means (with corresponding 95% confidence intervals) were calculated. Differences among categories were tested for statistical significance using Scheffé's adjustment for multiple comparisons.

## Results

Among the 195 countries, 130 that had complete data on PD for the years 2004-2006 were included for analyses. The 130 countries were randomly and equally split into the training set and the validation set. Descriptive statistics of the variables are shown in Table 1 and Table 2. By ANOVA, the descriptive data in the training and validation sets were found not significantly different. As PD was positively-skewed and the plot of standardized residuals versus predicted values showed increasing error variance, a square root transformation was used to stabilize the regression variance of PD.

**Table 1:** The means (standard deviation) of physician manpower-related variables in 130 countries, in training and validation sets

*Variables*	*Training Set(N = 65)*	*Validation Set(N = 65)*	*Entire Set(N = 130)*
Physician density(per 10,000)	15.2 (14.0)	17.6 (14.8)	16.4 (14.4)

Population density(per square kilometre)	135.7 (222.4)	137.2 (214.7)	136.4 (217.8)

Proportion under age 15 years	29.6 (12.0)	29.3 (11.0)	29.4 (11.5)

Proportion over age 60 years	11.4 (7.4)	10.6 (6.7)	11.0 (7.1)

Life expectancy (years)	65.5 (12.4)	66.4 (11.8)	65.9 (12.1)

Gross domestic product (GDP, per capita)	10177 (15811)	10091 (14279)	10134 (15005)

Expenditure on health as percentage of GDP	6.6 (2.7)	6.6 (2.8)	6.6 (2.8)

Purchasing power parities	438 (1518)	299 (924)	370 (1258)

*Notes*. Data are partitioned into two subsets in a cross-validation analysis. The "training set" is the subset used for analysis, and the "validation set" is used for validating the analysis

**Table 2:** The distribution of area (continent) and economic status in 130 countries, in training and validation sets

	Training Set (N = 65)	Validation Set (N = 65)	Entire Set (N = 130)
Continent			
Africa	26 (40.0)	18 (27.7)	44 (33·8)
Americas	3 (4.6)	1 (1.5)	4 (3.2)
Asia	7 (10.8)	5 (7.7)	12 (9.2)
Australia/Oceania	2 (3.1)	5 (7.7)	7 (5.4)
Europe	20 (30.7)	24 (36.9)	44 (33.8)
Middle East	7 (10.8)	12 (18.5)	19 (14.6)
Member of OECD			
No	50 (76.9)	50 (76.9)	100 (76.9)
Yes	15 (23.1)	15 (23.1)	30 (23.1)
Economics			
Low income	17 (26.1)	20 (30.8)	37 (28.4)
Middle income	28 (43.1)	25 (38.4)	53 (40.8)
High income	20 (30.8)	20 (30.8)	40 (30.8)

OECD = Organization for Economic Cooperation and Development

**Figure 1:**
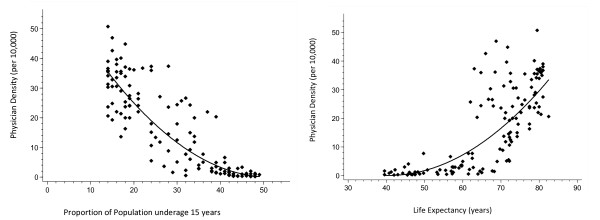
**(a) Relationship between physician density (y axis) and proportion of population under age 15 years (x axis)**. The regression line equation is: PD = (8.179 - 0.159 × PropPop)^2^. (**b**) Relationship between physician density (y axis) and life expectancy (x axis). The regression line equation is: PD = (-5.577 + 0.138 × LifeExp)^2^.

The stepwise regression procedure retained the same two variables in both the training and validation sets: proportion under age 15 years and life expectancy. The univariate relationships between PD and each retained predictor variable are illustrated in Figures 1a and 1b. The regression coefficients from the multivariate analyses are shown in Table 3. The R^2^s were virtually identical in both sets and none of the regression coefficients were statistically different between the two sets. The shrinkage coefficient was 1.5%, indicating a high level of model generalizability. Given a low level of shrinkage, the data were combined from both sets and a final regression equation was derived based upon the entire sample of 130 countries: PD = (5.014 - 0.128 × proportion under age 15 years + 0.034 × life expectancy)^2^. The R^2 ^of 80.4% from the final 2-variable model was virtually identical to the R^2 ^of 80.3% from a full model consisting of all 7 variables. (Note: The 7 predictor variables were: population density, proportion under age 15 years, proportion over age 60 years, life expectancy, gross domestic product, expenditure on health, and purchasing power parities).

**Table 3:** Regression coefficients (95% confidence intervals) from the Physician Density Prediction Model of two variables (proportion under age 15 years, life expectancy)

	*Training Set* *(N = 65)*	*Validation Set* *(N = 65)*	*Entire Set* *(N = 130)*
Intercept	4.841 (1.543, 8.139)	5.412 (2.056, 8.777)	5.014* (2.676, 7.351)
Proportion under age 15 years	-0.125 (-0.162, -0.088)	-0.134 (-0.172, -0.096)	-0.128* (-0.154, -0.102)
Life expectancy (years)	0.033 (-0.002, 0.068)	0.033 (-0.003, 0.069)	0.034^†^ (0.009, 0.059)
R-squared	81.6%	80.1%	80.4%

*Notes*. * P-value < 0.001; ^†^P-value = 0.007

**Figure 2:**
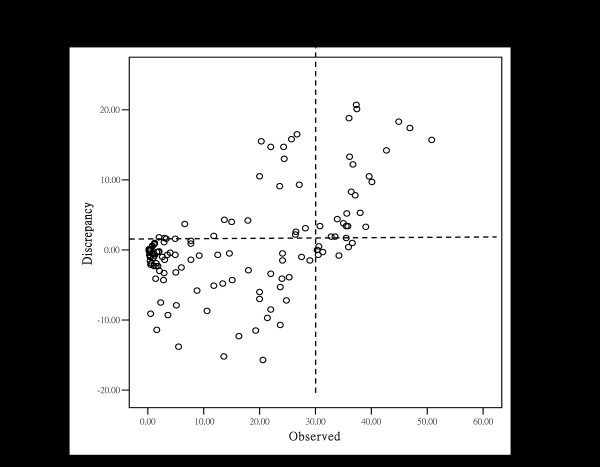
**Relationship between discrepancy between the predicted and the observed PD (y axis) and observed physician density (x axis)**. The breakpoint for "above the norm" in physician density occurs at approximately 30 per 10,000.

The present study used 2009 population data and the two model variables (the proportion under age 15 years and the life expectancy) to calculate a "predicted" (the norm) PD for a country. The "predicted" PD was then compared to the observed PD for each specific country, resulting in a calculated PD discrepancy. Table 4 ranks the countries from the highest to the lowest level of PD discrepancy. For a "negative discrepancy" (the observed PD less than the predicted PD), physician manpower can be considered as "under the norm", rather than "a deficit". In contrast, a "positive discrepancy" indicates the observed PD is greater than the predicted PD, and can be considered as "above the norm". There were 70 countries (70/130, 53.8%) with observed PDs that had "negative discrepancy", and 58 that had "positive discrepancy" (58/130, 44.6%). Figure 2 shows the relationship between PD discrepancy and observed PD in the 130 countries. It's interesting to note from Figure 2 that the breakpoint for "above the norm" in PD occurs at approximately 30 physicians per 10,000 population. The scatter-plot graph is divided into four quadrants by the vertical line of 30 PD and the horizontal line of "zero" discrepancy. Few countries with PD greater than 30 (per 10,000 population) were found to be "below the norm" in PD.

**Table 4:** The predicted and observed physician density (PD), continent, economic status, and analysis set in 130 countries, rank-ordered by the predicted-observed PD discrepancy

Rank	Country	Continent	OECD	Economic	Analysis Set	Predicted PD	Observed PD	Discrepancy (per 10,000)	Population	Physician number
1	Japan	AS	Yes	H	T	36.3	20.6	-15.7	126,804,433	-199,397

2	Bosnia/Herzegovina	EU	No	M	V	28.8	13.6	-15.2	4,621,598	-7,005

3	Sri Lanka	AS	No	M	T	19.3	5.5	-13.8	21,513,990	-29,629

4	Korea	AS	Yes	H	T	28.6	16.3	-12.3	48,636,068	-59,672

5	Romania	EU	No	M	T	30.8	19.3	-11.5	22,181,287	-25,600

6	Suriname	SA	No	M	T	13.0	1.6	-11.4	486,618	-555

7	Slovenia	EU	No	H	V	34.3	23.7	-10.7	2,003,136	-2,134

8	Canada	NA	Yes	H	T	31.1	21.4	-9.7	33,759,742	-32,794

9	Myanmar	AS	No	L	V	12.9	3.6	-9.3	48,137,741	-44,694

10	Bhutan	AS	No	M	T	9.6	0.5	-9.1	699,847	-635

11	Mauritius	AF	No	M	T	19.3	10.6	-8.7	1,294,104	-1,132

12	Poland	EU	Yes	M	T	30.5	22.0	-8.5	38,463,689	-32,643

13	Morocco	AF	No	M	T	13.0	5.1	-7.9	31,627,428	-24,973

14	Kiribati	AO	No	M	V	9.8	2.3	-7.5	115,401	-87

15	Croatia	EU	No	M	T	32.0	24.8	-7.2	4,486,881	-3,237

16	Serbia	EU	No	M	T	27.0	20.0	-7.0	7,344,847	-5,110

17	Montenegro	EU	No	M	V	26.0	20.0	-6.0	666,730	-399

18	Iran	ME	No	M	T	14.6	8.8	-5.8	67,037,517	-39,190

19	United Kingdom	EU	Yes	H	T	29.0	23.7	-5.3	61,284,806	-32,431

20	Albania	EU	No	M	V	16.9	11.8	-5.1	3,659,616	-1,859

21	Tunisia	AF	No	M	V	18.2	13.4	-4.8	10,589,025	-5,122

22	Seychelles	AF	No	M	T	19.4	15.1	-4.3	88,340	-38

23	Bangladesh	AS	No	L	V	7.1	2.8	-4.3	158,065,841	-68,214

24	Cyprus	EU	No	H	V	28.1	24.0	-4.1	1,102,677	-453

25	Vanuatu	AO	No	M	V	5.5	1.4	-4.1	221,552	-91

26	Luxembourg	EU	Yes	H	T	29.2	25.3	-3.9	497,538	-193

27	New Zealand	AO	Yes	H	V	25.4	22.0	-3.4	4,252,277	-1,436

28	Gabon	AF	No	M	V	6.2	2.9	-3.3	1,545,255	-509

29	Syria	ME	No	M	T	8.2	5.0	-3.2	22,198,110	-7,090

30	Nepal	AS	No	L	V	5.1	2.1	-3.0	28,951,852	-8,570

31	Kuwait	ME	No	H	V	20.9	18.0	-2.9	2,789,132	-822

32	India	AS	No	M	T	8.5	6.0	-2.5	1,180,512,215	-294,800

33	Djibouti	AF	No	M	T	4.1	1.8	-2.3	740,528	-174

34	Ghana	AF	No	L	V	3.8	1.5	-2.3	24,339,838	-5,699

35	Mauritania	AF	No	L	T	3.4	1.1	-2.3	3,205,060	-743

36	Eritrea	AF	No	L	T	2.6	0.5	-2.1	5,792,984	-1,195

37	Senegal	AF	No	L	T	2.5	0.6	-1.9	14,086,103	-2,691

38	Comoros	AF	No	L	V	3.4	1.5	-1.9	773,407	-143

39	Togo	AF	No	L	V	2.0	0.4	-1.6	6,199,841	-977

40	Macedonia	EU	No	M	V	25.6	24.1	-1.5	2,072,086	-320

41	Finland	EU	Yes	H	V	30.5	29.0	-1.5	5,255,068	-796

42	Namibia	AF	No	M	T	4.4	3.0	-1.4	2,128,471	-297

43	Nauru	AO	No	M	V	9.1	7.7	-1.4	14,264	-2

44	Timor-Leste	AS	No	M	T	2.1	1.0	-1.1	1,131,612	-129

45	Benin	AF	No	L	V	1.4	0.4	-1.0	9,056,010	-944

46	Sudan	AF	No	M	V	3.6	2.6	-1.0	41,980,182	-4,240

47	Australia	AO	Yes	H	T	28.5	27.5	-1.0	21,515,754	-2,165

48	Rwanda	AF	No	L	V	1.4	0.5	-0.9	11,055,976	-954

49	Germany	EU	Yes	H	V	35.0	34.2	-0.8	82,282,988	-6,898

50	Maldives	AS	No	M	V	10.0	9.2	-0.8	395,650	-31

51	Côte d'Ivoire	AF	No	L	T	2.0	1.2	-0.8	21,058,798	-1,639

52	Libya	AF	No	M	V	13.2	12.5	-0.7	6,461,454	-480

53	Cape Verde	AF	No	M	T	5.6	4.9	-0.7	431,822	-31

54	Mozambique	AF	No	L	T	1.0	0.3	-0.7	22,061,451	-1,531

55	Laos	AS	No	L	T	4.2	3.5	-0.7	6,993,767	-477

56	Hungary	EU	Yes	H	T	31.2	30.5	-0.7	9,880,059	-645

57	Guinea	AF	No	L	V	1.7	1.1	-0.6	10,324,025	-665

58	Central African Rep.	AF	No	L	T	1.3	0.8	-0.5	4,578,768	-231

59	United States	NA	Yes	H	T	24.6	24.1	-0.5	310,232,863	-14,835

60	Turkey	ME	Yes	M	V	15.0	14.6	-0.5	77,804,122	-3,674

61	Sierra Leone	AF	No	L	T	0.7	0.3	-0.4	5,245,695	-224

62	Burundi	AF	No	L	T	0.7	0.3	-0.4	9,863,117	-406

63	Botswana	AF	No	M	V	4.4	4.0	-0.4	2,029,307	-74

64	Latvia	EU	No	M	T	31.7	31.3	-0.3	2,217,969	-74

65	Cameroon	AF	No	M	T	2.2	1.9	-0.3	19,294,149	-626

66	Zimbabwe	AF	No	L	V	1.9	1.6	-0.3	11,651,858	-307

67	Congo (Brazzaville)	AF	No	M	V	2.2	2.0	-0.2	4,125,916	-71

68	Chad	AF	No	L	T	0.5	0.4	-0.1	10,543,464	-105

69	Malawi	AF	No	L	T	0.3	0.2	-0.1	15,447,500	-150

70	Burkina Faso	AF	No	L	T	0.5	0.5	0.0	16,241,811	-80

71	Ukraine	EU	No	M	T	30.3	30.3	0.0	45,415,596	-47

72	Liberia	AF	No	L	T	0.2	0.3	0.1	3,441,790	30

73	Niger	AF	No	L	V	0.1	0.2	0.1	15,306,252	170

74	Spain	EU	Yes	H	V	35.5	35.9	0.4	40,548,753	1,583

75	Angola	AF	No	M	V	0.3	0.8	0.5	13,068,161	704

76	Slovakia	EU	Yes	H	V	30.1	30.6	0.5	5,470,306	300

77	Mali	AF	No	L	T	0.2	0.8	0.6	13,796,354	844

78	Uganda	AF	No	L	V	0.2	0.8	0.6	33,398,682	2,044

79	Congo (Kinshasa)	AF	No	L	T	0.3	1.1	0.8	70,916,439	5,561

80	South Africa	AF	No	M	T	6.8	7.7	0.9	49,109,107	4,373

81	Zambia	AF	No	L	V	0.3	1.2	0.9	12,056,923	1,110

82	Guinea-Bissau	AF	No	L	T	0.2	1.2	1.0	1,565,126	151

83	Andorra	EU	No	H	V	35.6	36.6	1.0	84,525	8

84	Madagascar	AF	No	L	T	1.8	2.9	1.1	21,281,844	2,270

85	Pakistan	ME	No	L	V	6.4	7.7	1.3	177,276,594	22,940

86	Sao Tome & Principe	AF	No	L	V	3.3	4.9	1.6	219,334	36

87	Yemen	ME	No	L	V	1.7	3.3	1.6	23,495,361	3,871

88	Equatorial Guinea	AF	No	H	T	1.3	3.0	1.7	650,702	108

89	Czech Republic	EU	Yes	H	T	33.8	35.5	1.7	10,201,707	1,726

90	Afghanistan	ME	No	L	V	0.2	2.0	1.8	29,121,286	5,274

91	Estonia	EU	No	H	T	30.9	32.8	1.9	1,291,170	249

92	Portugal	EU	Yes	H	V	31.6	33.5	1.9	10,735,765	2,081

93	Cook Islands	AO	No	H	T	9.8	11.8	2.0	23,000	5

94	Qatar	ME	No	H	V	24.2	26.4	2.2	840,926	186

95	Moldova	EU	No	M	V	23.9	26.5	2.6	4,320,748	1,137

96	Ireland	EU	Yes	H	V	25.0	28.2	3.1	4,250,163	1,331

97	Italy	EU	Yes	H	T	35.7	39.0	3.3	58,090,681	19,097

98	Austria	EU	Yes	H	V	32.1	35.5	3.4	8,214,160	2,762

99	Bulgaria	EU	No	M	T	32.3	35.8	3.4	7,148,785	2,436

100	Denmark	EU	Yes	H	T	27.4	30.8	3.4	5,515,575	1,897

101	Iraq	ME	No	M	V	2.9	6.6	3.7	29,671,605	11,054

102	Sweden	EU	Yes	H	T	31.2	35.0	3.8	9,059,651	3,465

103	Oman	ME	No	H	T	11.0	15.0	4.0	3,525,875	1,398

104	Mexico	NA	Yes	M	V	13.7	17.9	4.2	112,468,855	47,141

105	Saudi Arabia	ME	No	H	V	9.4	13.7	4.3	29,207,277	12,531

106	France	EU	Yes	H	V	29.5	33.9	4.4	64,768,389	28,812

107	Malta	EU	No	H	T	30.4	35.6	5.2	406,771	212

108	Switzerland	EU	Yes	H	V	32.7	38.0	5.3	7,623,438	4,026

109	Netherlands	EU	Yes	H	V	29.3	37.1	7.8	16,783,092	13,090

110	Norway	EU	Yes	H	T	28.1	36.4	8.3	4,676,305	3,892

111	Lebanon	ME	No	M	T	14.5	23.6	9.1	4,060,766	3,689

112	Bahrain	ME	No	H	T	17.8	27.1	9.3	738,004	686

113	Belgium	EU	Yes	H	T	30.4	40.1	9.7	10,423,493	10,124

114	Lithuania	EU	No	M	T	29.1	39.6	10.5	3,545,319	3,725

115	Niue	AO	No	M	V	9.5	20.0	10.5	1,398	1

116	Iceland	EU	Yes	H	V	24.5	36.7	12.2	308,910	377

117	Kyrgyzstan	ME	No	L	V	11.4	24.4	13.0	5,508,626	7,183

118	Armenia	EU	No	M	V	22.9	36.1	13.3	2,966,802	3,933

119	Russia	EU	No	M	V	28.5	42.7	14.2	139,390,205	197,550

120	Jordan	ME	No	M	T	7.3	22.0	14.7	6,407,085	9,391

121	Egypt	AF	No	M	T	9.6	24.3	14.7	80,471,869	118,113

122	Tajikistan	ME	No	L	V	4.8	20.3	15.5	7,487,489	11,635

123	Greece	EU	Yes	H	V	35.1	50.8	15.7	10,749,943	16,836

124	Turkmenistan	ME	No	M	T	9.9	25.7	15.8	4,940,916	7,783

125	Uzbekistan	ME	No	L	V	10.2	26.7	16.5	27,865,738	45,950

126	Belarus	EU	No	M	V	29.5	46.9	17.4	9,612,632	16,757

127	Georgia	EU	No	M	T	26.5	44.9	18.3	4,600,825	8,442

128	Azerbaijan	EU	No	M	V	17.2	36.0	18.8	8,303,512	15,637

129	Israel	ME	No	H	V	17.3	37.4	20.1	7,353,985	14,787

130	Kazakhstan	AS	No	M	V	16.7	37.3	20.7	15,460,484	31,933

*Notes*. PD = physician density (per 10,000 population)

Discrepancy = Predicted PD minus Observed PD

Physician Number = Discrepancy × Population ÷ 10,000

T and V: T (Training set) or V (Validation set)

Continent: *AF *= Africa, *AS *= Asia, *AO *= Australia/Oceania, *EU *= Europe,*ME *= Middle East, *NA *= North America, *SA *= South America

*OECD *= Organization for Economic Cooperation and Development

Economics: L (Low income); M (Middle income); H (High income)

Statistically significant differences in PD discrepancies were observed when more "broad stroke" comparisons were made among the countries' continents, membership in the OECD, and economic levels (Table 5). In general, countries grouped within continents which were more 'westernized' (Americas and Europe) had a greater mean "deficit" of physicians (-4.3 and -6.3 physicians per 10,000) than other continents. This result was congruent with being a member of the OECD and being considered a high income country.

**Table 5:** Comparison of physician density (per 10,000) discrepancies by continent, OECD status, and economics from analysis of covariance

	Least Squares Mean Discrepancy (95% Confidence Interval)	P-value
Continent		
Africa (AF)	6·7 (4·6, 8·8)	< 0·001*
Americas (AM)	-4·3 (-9·3, 0·8)	
Asia (AS)	-1·2 (-4·1, 1·7)	
Australia/Oceania (OA)	1·1 (-2·7, 4·9)	
Europe (EU)	-6·3 (-8·6, -4·0)	
Middle East (ME)	5·8 (3·5, 8·2)	
Member of OECD		
No	2·3 (1·1, 3·5)	< 0·001
Yes	-4·1 (-6·6, -1·7)	
Economics		
Low income (L)	6·7 (4·5, 8·9)	< 0·001^†^
Middle income (M)	0·8 (-0·7, 2·3)	
High income (M)	-4·5 (-6·5, -2·4)	

OECD = Organization for Economic Cooperation and Development

* Statistically significant differences between AF & AM, AF & AS, AF & EU, AM & ME, AS & ME, EU & ME

^† ^Statistically significant differences between L & M, L & H, M & H

## Discussion

Physician density itself is not the only factor determining health outcomes. Evaluating physician manpower for appropriateness is a complex task that should simultaneously consider many influencing factors on population, physician productivity, healthcare system, and the economics. This study developed an international PD regression model that applied "Crowd Theory", which incorporated many factors affecting observed PDs in 130 countries. The "predicted PDs", derived from the regression model was used as the "norm" for the PD that countries commonly used in maintaining their healthcare.

The final regression model retained two variables (proportion under age 15 years and life expectancy), which accounted for 80.4% of the total variance of PD. In predicting PD, "proportion under age 15 years" had an inverse relationship, while "life expectancy" had a positive relationship. However, correlational relationships do not imply that one factor causes the other. Children under age 15 years actually tend to utilize more medical services than the average age, thus it was assumed that the larger the child population a country had, the more physicians were needed. The inverse relationship may have been due to a hidden third variable, such as a country's economics. Kwame found quantitative evidence on the relationship between certain socioeconomic and demographic factors (e.g., birth rate, being reflective of population under 15 and per capita health care expenditure in Africa (Kwame 1992) [15]. An inverse relationship was also reported between birth rates and the economy that was known positively correlated with physician density [16,17]. "Life expectancy", referring not only the length but also the quality of life, has been recognized as a standard measure in the world for measuring population health [18]. Therefore, it is of no surprise to have "life expectancy" retained in the regression model.

The relationship between physician density and healthcare quality has been a long time research focus. The shortage of physician manpower will increase physician workloads and hamper patient safety. The criterion for determining appropriate physician number is the level that physicians can provide acute healthcare and guarantee patient safety in a hospital setting. For example, in pediatrics, patient care with safety consideration is the care delivered to all children who visit, stay or are born in a hospital, day and night, attended by health care providers under reasonable workloads. Besides patient safety, when added to the requirements of timeliness, effectiveness, efficiency, equitableness and patient-centered care [19], physicians' workloads often increase and shortage of physician manpower emerges. It has been reported that heavy workloads and stress significantly impacts on patient care quality, physician performance, absenteeism, turnover and organizational performance [20]. Adequate physician manpower is one of the strategies to prevent physicians from burnout.

Early detection of physician shortage has been a great challenge. Soon after 2000 when physician supply was considered to exceed demand [21], physician shortages emerged in the United States and Canada [4]. Similar reports of physician shortages have also been reported in other developed countries, such as Japan [22,23], Australia and Singapore [24]. These countries have increased student enrollment and established new medical schools to overcome the deficit. Unfortunately, the correction of physician shortage within a country has been taking an extended period of time, e.g., nearly 25 years in the United States.

The regression model in this study is derived from data of 130 countries around the world that reflects the PD most in a country. Thus, the predicted PD would be better used as a warning sign rather than an absolute number suggested for correction. A discrepancy between the predicted and the observed PD in a country indicates the physician manpower is either in surplus or in deficit, deviated from the norm of crowd behavior. A large negative discrepancy will highlight the needs to survey their physician's workloads and their well-being, and to improve quality of medical performance as well.

There seems to be a potential to increase physician number to improve health outcome, especially when physician shortages became a global concern. As early as 1986, Perrin reported simply increasing physician supply may not have much effect on healthcare quality [6]. To improve health status, benefits will also come from the focus on improving adherence to the standards of best medicine and from preventive efforts to diminish personal risk factors of disease (smoking, diet, and exercise). More focused efforts should also be put to improve physicians' competency, the services that they provide, and the team resource management, rather than just increasing physician supply [6,25]. The above factors, being difficultly translated into "data" for analyses, may explain why some countries with negative discrepancy of PD had good health outcome.

## Conclusion

An appropriate size of physician workforce is vital to maintain a nation in good health. When facing the crisis of physician shortage, the correction of manpower always takes an extended time period, and thus both the public and health personnel suffer. The regression PD model which provides information on how the observed PD deviates from the "norm" can be used to screen the appropriateness of physician manpower in a nation. To prevent damage to healthcare system when discrepancy appears between the observed and the predicted PD, we should examine not only the status of physician manpower, but also the physicians' workloads, the quality of medical performance, the physicians' well-being, the effectiveness of health promotional program and the team resource management.

## Competing interests

The authors declare that they have no competing interests.

## Authors' contributions

TC established the research design, carried out the data collection, participated in data interpretation and composed the manuscript. ME did data analyses, revised the manuscript, and gave final approval. DF participated in the literature reviews, provided concepts of the research design, and revised the manuscript. All authors read and approved the final manuscript.

## Pre-publication history

The pre-publication history for this paper can be accessed here:

http://www.biomedcentral.com/1472-6963/12/79/prepub
